# Importance of residue 248 in *Escherichia coli* RNase P RNA mediated cleavage

**DOI:** 10.1038/s41598-023-41203-4

**Published:** 2023-08-29

**Authors:** Guanzhong Mao, Abhishek S. Srivastava, Shiying Wu, David Kosek, Leif A. Kirsebom

**Affiliations:** Department of Cell and Molecular Biology, Biomedical Centre, Box 596, 751 24 Uppsala, Sweden

**Keywords:** Molecular biology, Ribozymes

## Abstract

tRNA genes are transcribed as precursors and RNase P generates the matured 5' end of tRNAs. It has been suggested that residue − 1 (the residue immediately 5ʹ of the scissile bond) in the pre-tRNA interacts with the well-conserved bacterial RNase P RNA (RPR) residue A_248_ (*Escherichia coli* numbering). The way A_248_ interacts with residue − 1 is not clear. To gain insight into the role of A_248_, we analyzed cleavage as a function of A_248_ substitutions and N_−1_ nucleobase identity by using pre-tRNA and three model substrates. Our findings are consistent with a model where the structural topology of the active site varies and depends on the identity of the nucleobases at, and in proximity to, the cleavage site and their potential to interact. This leads to positioning of Mg^2+^ that activates the water that acts as the nucleophile resulting in efficient and correct cleavage. We propose that in addition to be involved in anchoring the substrate the role of A_248_ is to exclude bulk water from access to the amino acid acceptor stem, thereby preventing non-specific hydrolysis of the pre-tRNA. Finally, base stacking is discussed as a way to protect functionally important base-pairing interactions from non-specific hydrolysis, thereby ensuring high fidelity during RNA processing and the decoding of mRNA.

## Introduction

The tRNA genes are transcribed as precursors (pre-tRNA) and several enzymes are involved in the processing of pre-tRNA. Among these endoribonuclease P (RNase P) is responsible for generating tRNAs with matured 5ʹ ends. RNase P from all three kingdoms of life consists of both RNA and protein; in Bacteria, RNase P is composed of one RNA (RPR) and one protein subunit, C5. Irrespective of origin, the catalytic activity was thought to reside in the RPR^1,2^. Recent data, however, show that there exist RNase P activities solely based on proteins, (referred to as PRORPs) in human mitochondria, *Arabidopsis thaliana*, *Trypanosoma brucei*, in the algae *Ostreococcus tauri* and in *Aquifex aeolicus*^3–7^.

**Figure 1:**
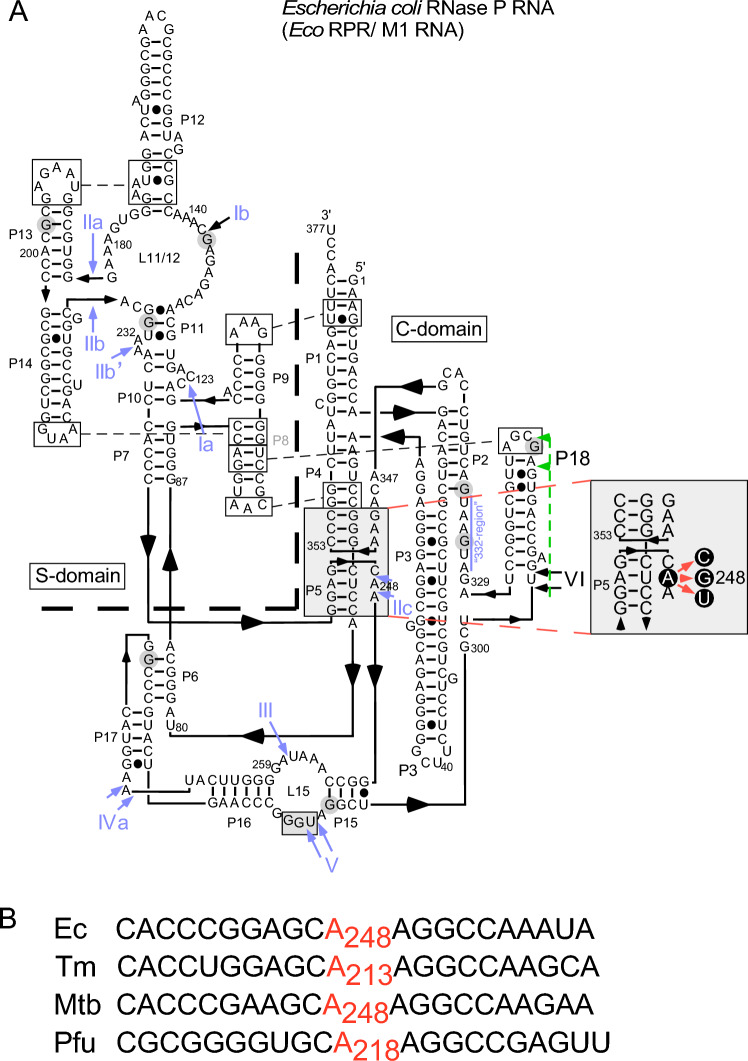
Illustration of the *Eco* RPR secondary structures. (**A**) *Eco* RPR secondary structure according to Massire et al.^90^. The heavy dashed demarcation line separates the S- and C-domains. The large gray box highlights the A_248_-region, and show the substitutions that were introduced at 248 (red arrows). The gray box in L15 marks residues that pair with the substrate 3ʹ end—the RCCA-RNase P RNA interaction (interacting residues underlined)^12^—in the RPR-substrate complex. The blue arrows and Roman numerals mark the Pb^2+^-induced cleavage sites as shown in Fig. 2 (black circles). The vertical line marked in blue marks the "332-region", which is also cleaved in the in presence of Pb^2+^(see also^85,91^). Residues highlighted with gray circles correspond to RNase T1 cleavage sites (see also Fig. 2, bands marked with red dots)^92^. The green dashed line and arrows mark the area in P18, which becomes accessible to RNase T1 cleavage upon on substitution of A_248_ with U (see Fig. 2, *Eco* RPR_U248_). (**B**) Sequence of alignment of the region which includes the conserved *E. coli* (Ec) A_248_, *T. maritima* (Tm^93^) A_213_, *M. tuberculosis* (Mtb^28^) A_248_ and the Archaea *P. furiosus* (Pfu^9,12^) A_218_, and neighboring sequences as indicated.

At high ionic strength, RPRs of different origins cleave pre-tRNA and a number of non-tRNA substrates efficiently at the correct site without proteins^1,8–12^. The RPR interacts with several regions of pre-tRNAs and model substrates. These are: the 3ʹ terminal RCC-motif (the RCCA-RNase P interaction, interacting residues underlined)^13^; and the T-stem/loop- (TSL) region of pre-tRNAs binds to the RPR TSL-binding site or TBS in the specificity (S) domain. In addition, the residue immediately 5ʹ of the cleavage site (N_−1_) is in close proximity to A_248_ (referred to as the A_248_/N_−1_ interaction and *Escherichia coli* numbering; Fig. 1). The crystal structure of bacterial (*Thermotoga maritima*) RNase P in complex with tRNA and recent cryo-EM structures of yeast, archaeal and human RNase P in complex with pre-tRNA (yeast) and tRNA suggest that in particular the TSL–TBS interaction is evolutionary conserved^11,14–21^, (see also^22^).

Residue A_248_ is well-conserved among bacterial RPRs. However, the nature of the A_248_/N_−1_ interaction is less clear. In the bacterial RNase P-tRNA co-crystal structure A_248_ is positioned close to the tRNA 5ʹ end^18^. This is also observed in the cryo-EM structure of an archaeal RNase P in complex with tRNA^20^. On the basis of biochemical and genetic data using *E. coli* (*Eco*) RPR, A_248_ has been proposed to form a *cis* Watson–Crick/Watson–Crick (*cis* WC/WC)^23^ pair with the N_−1_ residue in the substrate^24,25^. The identity of residue −1 in pre-tRNAs varies in *E. coli* and other bacteria, but in many of them it is a uridine^26–28^. We have provided data that this pairing does not correspond to a standard Watson–Crick pairing. Rather, the N_−1_ residue binds to a pocket where A_248(wt)_ plays a central role, but it does not directly pair with the −1 residue^16,29–31^. Nucleotide analogue-modification interference studies further suggest that the Hoogsteen surface of A_248_ is important for a productive interaction with the substrate^32^. However, kinetic data argue against that N7 and 6NH_2_ of A_248_ form hydrogen bonds with the 2ʹOH and O2 (when present on the nucleobase, i.e. C or U) of residue −1, respectively, which both are oriented in the same direction in a structural model of the cleavage site^31^. Thus, the function of A_248_—and whether it interacts with residue –1—remains unclear. The crystal and cryo-EM structures of the RNase P-tRNA complexes (bacteria and archaea) do not provide guidance because these structures represent the post cleavage stage of the RNase P catalyzed reaction^18,20^. We therefore decided to revisit and investigate the interrelationship between residue −1 and A_248_. To achieve this, we studied cleavage of all ribo pre-tRNA and model hairpin loop substrates, carrying different nucleobases at position −1 with *Eco* RPR 248-variants.

Here we provide data that the identities of both residue N_−1_ in the substrate and residue 248 in the RPR influence cleavage site selection and rate of cleavage. However, our data do not support the model where the well-conserved residue A_248(wt)_ forms a *cis* WC/WC pair with N_−1_. This was particularly apparent studying different substrates carrying 3-methyl U at the N_−1_ position. Our combined data support a model where the structural topology of the active site varies and depends on the identity of the nucleobases at, and in proximity to, the cleavage site and their potential to interact. As a consequence, this affects the positioning of Mg^2+^ that activates the water that acts as the nucleophile resulting in efficient and correct cleavage. In this scenario we suggest that, besides participating in the anchoring of the substrate, the role of A_248_ in wild type bacterial RPR, which stacks on the tRNA G_+1_/C_+72_ base pair, is to exclude bulk water from accessing the amino acid acceptor stem and thereby prevent non-specific hydrolysis/cleavage of the pre-tRNA.

## Results

### Substituting residue 248 has minor effects on the overall structure of Eco RPR

To investigate the role and contribution of the well-conserved A_248_ to *Eco* RPR mediated cleavage we used wild type *Eco* RPR_A248(wt)_ and three 248 variants: *Eco* RPR_C248_, *Eco* RPR_G248_ and *Eco* RPR_U248_ (Fig. 1). The generation and catalytic performance of *Eco* RPR_G248_ has been reported elsewhere^30,31^, while the other two RPRs were generated as outlined in “Materials and methods”. As predicted on the basis of previous studies, the C_248_ and U_248_ variants were catalytically active^24^ (see below).

**Figure 2:**
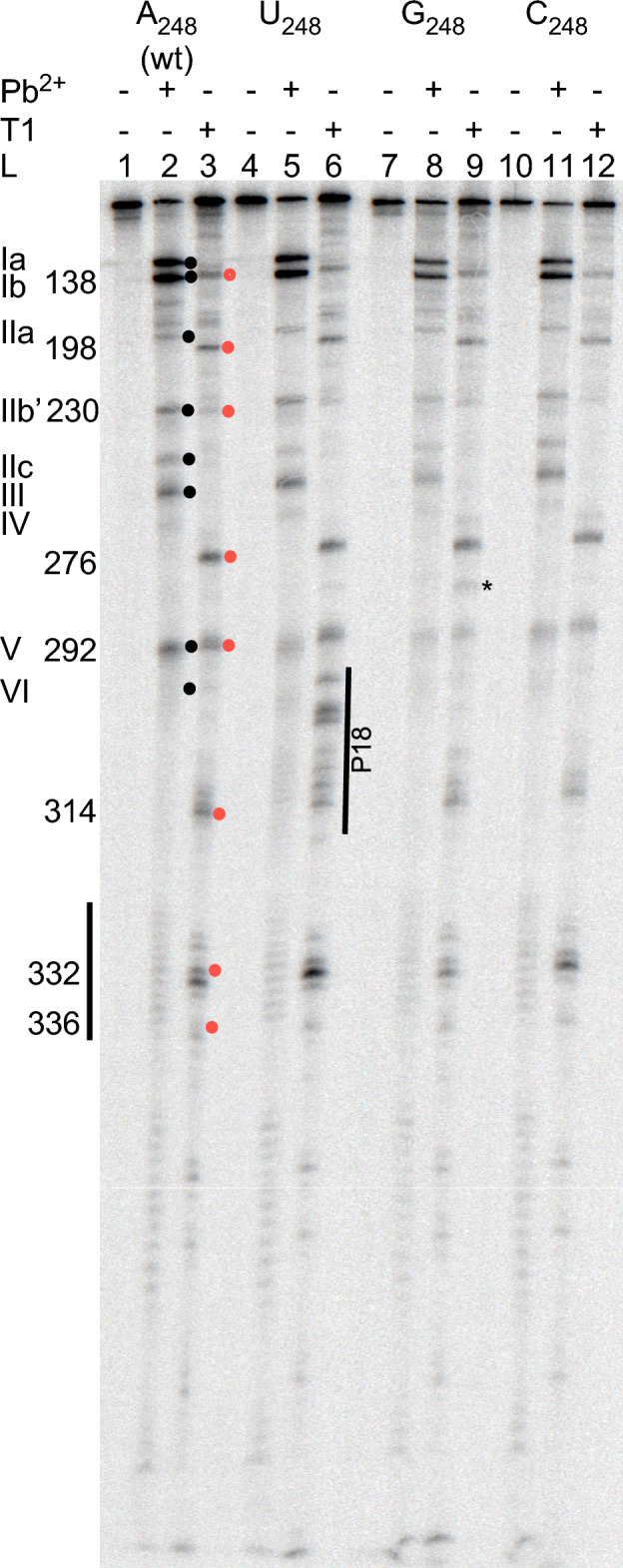
Structural probing of *Eco* RPR. Probing the structures of the *Eco* RPR 248-variants with Pb^2+^ and RNase T1. Roman numerals and black circles refer to Pb^2+^-induced cleavage sites in *Eco* RPR (Fig. 1)^31,85,91^. Numbers and red circles correspond to the RNase T1 cleavage sites according to Guerrier-Takada and Altman^92^, see Fig. 1A. The vertical black lines mark the P18- and 332-region. The vertical black line "P18" marks the extra RNase T1 cleavage sites between 292 and 314 in the U_248_ variant. The reactions were conducted using 0.5 mM Pb(OAc)_2_ and RNase T1 as described in “Materials and methods”.

First, we inquired whether substitution of A_248(wt)_ with any of the other nucleobases affected the structure of *Eco* RPR. On the basis of structural probing with Pb^2+^ and RNase T1 (which cleaves 3ʹ of single stranded G residues) we reported that the overall structures of *Eco* RPR_A248(wt)_ and *Eco* RPR_G248_ are very similar^31^. This was also the case for the C_248_ variant [Fig. 2; cf. lanes 2 and 3 (*Eco* RPR_A248(wt)_) and lanes 11 and 12 (*Eco* RPR_C248_)]. By contrast, a U at 248 affected the structure such that G-residues between P15 and the P18-loop became accessible to RNase T1 [Fig. 1; cf. lanes 3 (*Eco* RPR_A248(wt)_) and 6 (*Eco* RPR_U248_)]. This suggested that a U at 248 influences the structural integrity of P18, which plays a role in connecting the C- and S-domain via the P8/P18-interaction (Fig. 1). Moreover, compared to *Eco* RPR_A248(wt)_, exposure to RNase T1 resulted in the appearance of an additional weak cleavage product located between residues 276 and 292, in particular in the case of G_248_ (Fig. 2; bands marked with *). This might indicate a change in the structure in this region in response to mutating A_248(wt)_, see also Ref.^31^. With respect to the Pb^2+^-induced cleavage patterns, we did not detect any apparent difference comparing the 248 variants [Fig. 2; cf. lanes 2 (A_248(wt)_), 5 (U_248_), 8 (C_248_) and 11 (G_248_)]. We conclude that substitution of A_248_ in wild type *Eco* RPR resulted in a small (if any) overall structural effect with the exception of U_248_ where a notable structural change was detected in P18.

### Catalytic performance as a function of replacing the well-conserved A_248_ in *Eco* RPR

#### Choice of substrates and experimental outline

**Figure 3:**
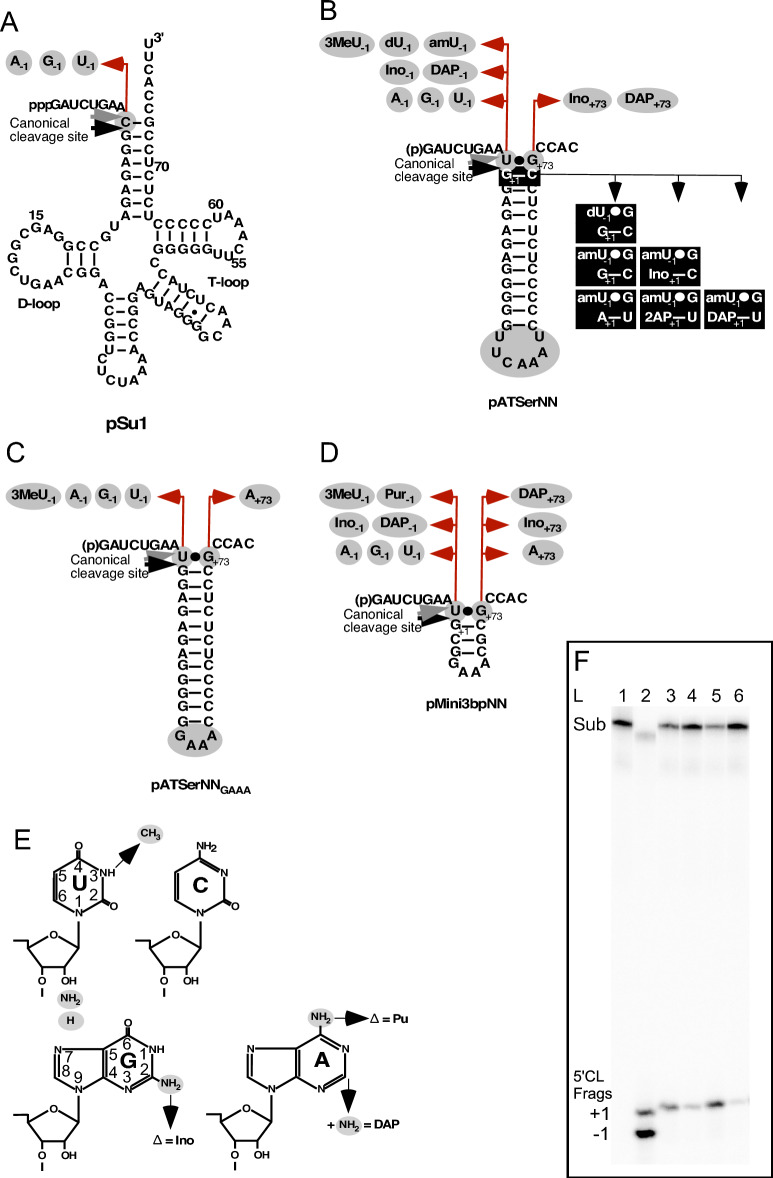
Secondary structure of substrates used in the present study. (**A**) pSu1, (**B**) pATSerNN, (**C**) pATSerNN_GAAA_, (**D**) pMini3bp, (**E**) structures of nucleobases, and (**F**) cleavage of pATSerUG by the different *Eco* RPR 248 variants. Residues highlighted in gray were introduced to generate the different variants carrying alternative nucleobases at positions −1 and +73. The black boxes illustrate the changes that generated substrates carrying 2ʹNH_2_ and 2ʹH as well as substitutions of residues at positions +1 and +72. The canonical (correct) cleavage sites between residues N_−1_ and N_+1_ in the different substrates are marked with black arrows. The gray arrows mark the alternative cleavage sites between N_−2_ and N_−1_ (referred to as position −1, see text). The seven-base loop (**B**, marked in gray) in pATSerNN was replaced with a GAAA-tetra loop (**C**, marked in gray) to generate pATSerNN_GAAA_, see^14,15^. Panel (**F**): lane (L) 1, pATSerUG no RPR added; lane 2, cleavage of pATSerCG_GAAA_ with *Eco* RPR_A248(wt)_; lane 3, cleavage of pATSerUG with *Eco* RPR_A248(wt)_; lane 4, cleavage of pATSerUG with *Eco* RPR_C248_; lane 5, cleavage of pATSerUG with *Eco* RPR_G248_; lane 6, cleavage of pATSerUG with *Eco* RPR_U248_. Sub, substrate and 5ʹCL Frags marks the migration of the 5ʹ cleavage products as a result of cleavage at +1 and −1. The reaction was performed in buffer C at 800 mM Mg^2+^ with 0.8 μM *Eco* RPR (irrespective of variant) and ≤ 0.02 μM substrate for 10 s as described in “Materials and methods”.

The role of residues A_248_ in *Eco* RPR_A248(wt)_ and −1 in the substrate has previously been analyzed using variants of a *Bacillus subtilis* tRNA^Asp^ precursor^24,25^. From these studies, the authors proposed a model where the −1 residue in the substrate forms a *cis* Watson–Crick (WC) base pair with A_248_. The model predicts that (i) breakage of this interaction shifts cleavage from the correct to an alternative site (see below), and (ii) introduction of a compensatory change that restores the N_−1_/N_248_ pairing should increase (rescue) cleavage at the correct site. To test this model and to investigate the role of A_248_, we used N_−1_ derivatives of the *E. coli* tRNA^Ser^Su1 precursor, pSu1^13,33^, and two well-characterized model hairpin loop substrates, pATSer and pMini3bp, both derived from pSu1 (Fig. 3)^15,31,34–36^. The pATSer substrates have the amino acceptor-stem and T-stem intact while pMini3bp lacks the T-stem, T-loop and part of the acceptor-stem. Two pATSer variants were used, the first has the original T-loop (e.g. pATSerUG where U and G correspond to the residues at −1 and " +73", respectively; numbering refers to the position in tRNA; Fig. 3). In the other, the T-loop is substituted with a GAAA-tetra loop (e.g. pATSerUG_GAAA_). The latter interacts differently with *Eco* RPR_A248(wt)_; it increases cleavage at the alternative site between −2 and −1 (Fig. 3C; see below)^15,30,37^. The short model substrates pMini3bp all have three-base-pair short stems, capped with GAAA-tetra loops (e.g. pMini3bpUG). Importantly, pSu1 and pATSer can interact with the TBS-region (see above) upon *Eco* RPR substrate complex formation, while pATSer variants with GAAA-tetra loops and pMini3bp cannot (or interact differently) due to their sizes and/or the presence of the GAAA-tetra loop^15,30,31,38^. We introduced the natural ribonucleobases (A, C, G and U) at position −1 (N_−1_) in all four substrate variants. For pATSer and pMini3bp, we also used variants carrying chemically modified ribonucleobases at −1 and +73. Varying both residue −1 and +73 allowed us to investigate the importance of having nucleobases at −1 that can pair with residue +73 with different numbers of hydrogen bonds. To further investigate whether U_−1_ in the model substrates pairs with A_248(wt)_ in *Eco* RPR we introduced a methyl group (**3mU**) at − 1 (Fig. 3E), which interferes with *cis* WC/WC pairing with A_248(wt)_. Finally, we replaced the 2ʹOH with 2ʹNH_2_ (or 2ʹH) and varied the +1/+72 base pair in pATSerUG to probe the de-protonation of the 2ʹNH_2_ (charge distribution; see below) at the canonical cleavage site in the RPR-substrate complex as a function of N_248_ identity (Fig. 3)^38–40^.

The Mg^2+^ concentration for optimal cleavage rates of pMini3bp substrates using *Eco* RPR_A248(wt)_ and *Eco* RPR_G248_ is 800 mM; this is higher than for the other substrates^15,30,31^. Moreover, on the basis of our published data where we studied cleavage of pATSer and pMini3bp variants using *Eco* RPR_A248(wt)_ and *Eco* RPR_G248_, we assumed that optimal cleavage rates are reached at 800 mM Mg^2+^ also for the other 248 variants^15,30,31,37^. To be able to directly compare the cleavage rates, we decided to perform all the experiments discussed below at 800 mM Mg^2+^. Also, at this Mg^2+^ concentration the likelihood of detecting cleavage increases, see e.g.,^31^. We emphasize that the C5 protein interacts with residues N_−4_–N_−8_ in the 5ʹ leader but not N_−1_^41,42^ and that we were primarily interested in the catalytic performance of the RPR in the absence of C5. Hence, these studies were performed without the C5 protein.

**Figure 4:**
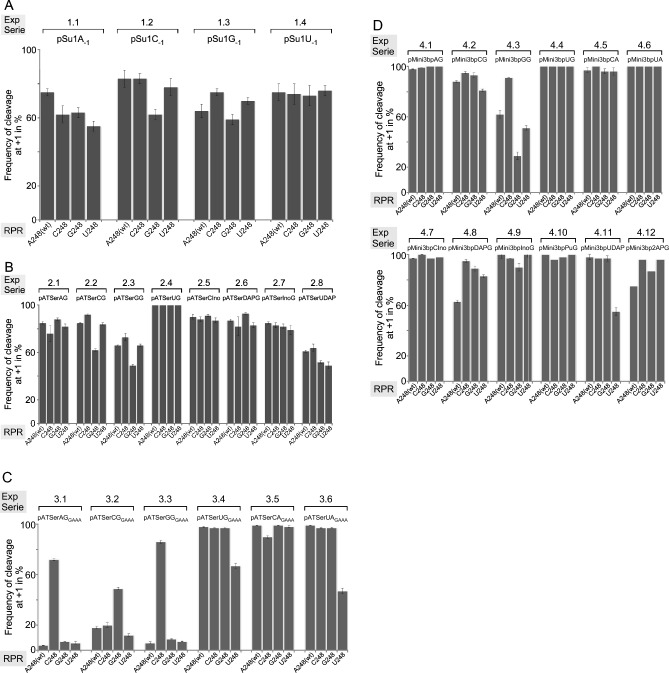
Frequencies of cleavage at +1 by *Eco* RPR 248 variants. Histograms summarizing frequencies of cleavage at +1 in % for the various substrate and *Eco* RPR 248 combinations as indicated. (**A**) Cleavage of pSu1(N_−1_) variants, Exp Series (ExpS) 1.1–1.4. (**B**) Cleavage of pATSer(N_−1_N_+73_) variants, Exp Series (ExpS) 2.1–2.8. (**C**) Cleavage of pATSer(N_−1_N_+73_)_GAAA_ variants, Exp Series (ExpS) 3.1–3.6. (**D**) Cleavage of pMini3bpN_−1_/N_+73_ variants, Exp Series (ExpS) 4.1–4.12. To calculate the frequencies of cleavage at +1 we used the 5ʹ cleavage fragments and mean and experimental errors were calculated from at least three independent experiments.

Cleavage of the different substrates was studied with respect to (i) cleavage site recognition and (ii) rate of cleavage (single turnover; see “Materials and methods”). The canonical (also referred to as correct cleavage or the +1 position) site corresponds to cleavage between residues −1 and +1 (Fig. 3), while cleavage at other positions are referred to as alternative sites or miscleavage; e.g., cleavage at −1 relates to cleavage between −2 and −1 in the 5ʹ leader. The frequencies of cleavage at +1 are presented in Figs. 4, 6 and 8 while the rate constants (k_app_), determined under single turnover conditions for the combinations discussed above, are shown in Tables 1, 2 and 3. For clarity and guidance, the experiments using different substrate/RPR (N_248_) combinations are referred to as "Experiment Series (ExpS)" in the figures and tables where 1.1 corresponds to substrate 1, pSu1, having A at −1 while 1.2 has C, i.e. pSu1A_−1_ and pSu1C_−1_, respectively, and substrate 2.1 pATSer having A_−1_ and G_+73_ (pATSerAG and A_−1_/G_+73_ substrate/variant) etc. In the first set of experiments we studied cleavage of the full-size pre-tRNA^Ser^Su1 (pSu1; ExpS 1.1–1.4) and pATSer N_−1_/N_+73_ (ExpS 2.1–2.8; Fig. 4A,B, and Table 1) variants that can interact productively with the TBS in the RPR S-domain (see above). These results are discussed below in “Substrates that can interact productively with the TBS-region—influence of changes of the nucleobase at −1 in substrates and residue 248 in the RPR”. Following this we analyzed the impact of N_−1_/N_+73_ variants in substrates that cannot form a productive interaction with the TBS, pATSer_GAAA_ (ExpS 3.1–3.6; Fig. 4C and Table 1) and pMini3bp (ExpS 4.1–4.12; Fig. 4D and Table 2) variants. These data are discussed in “Substrates that cannot interact productively with the TBS-region—influence of changes of the nucleobase at −1 in substrates and residue 248 in the RPR”. In “Altering the WC-surface of a U at position −1 and influence of the N248 identity”, we discuss substrates carrying CH_3_ at position 3 on the nucleobase that alter the WC-surface of U_−1_ in model substrates (ExpS 5.1–5.3; Fig. 6 and Table 3). In each sections (A-C), we summarize the data with respect to the possible interaction between residues A_248_ and −1.

**Table 1:** Rate of cleavage (k_app_) for pSu1, pATSer and pATSer_GAAA_ derivatives using different RPR variants without the C5 protein.

	ExpS	CL Site	A_248_ wt	RPR variant		
				C_248_	G_248_	U_248_
pSu1A_−1_	1.1	+ 1	47 ± 3	32 ± 1	58 ± 5	38 ± 7
		− 1	3.5 ± 0.6	3 ± 0.4	4 ± 0.5	8 ± 0.7
pSu1C_−1_	1.2	+ 1	71 ± 7	44 ± 5	43 ± 4	46 ± 3
		− 1	5 ± 0.1	2.5 ± 0.2	17 ± 1	5 ± 1
pSu1G_−1_	1.3	+ 1	41 ± 2	44 ± 4	39 ± 0.7	36 ± 4
		− 1	6 ± 0.6	2 ± 0.2	12 ± 1	4 ± 0.3
pSu1U_−1_	1.4	+ 1	**121 ± 4**	70 ± 3	85 ± 3	47 ± 3
		− 1	4 ± 0.4	3 ± 0.1	4 ± 0.5	3 ± 0.3
pATSerAG	2.1	+ 1	64 ± 6.9	4.3 ± 0.2	**90 ± 6.4**	36 ± 3.4
		− 1	8 ± 2	0.8 ± 0.1	10 ± 1	6 ± 0.8
pATSerCG	2.2	+ 1	51 ± 1.2	10 ± 0.2	11 ± 0.5	24 ± 0.5
		− 1	9 ± 0.7	0.9 ± 0.1	7 ± 0.5	4 ± 0.2
pATSerGG	2.3	+ 1	40 ± 4.4	26 ± 1	48 ± 2.5	28 ± 6
		− 1	15 ± 3	7 ± 0.5	41 ± 2	11 ± 2
pATSerUG	2.4	+ 1	73 ± 1	32 ± 3	61 ± 0.4	11 ± 0.2
		− 1	ND	ND	ND	ND
pATSerCIno	2.5	+ 1	47 ± 5	17 ± 2.2	61 ± 11	19 ± 0.1
		− 1	4 ± 0.4	2 ± 0.4	5 ± 1	2 ± 0.1
pATSerDAPG	2.6	+ 1	47 ± 1	4 ± 1	72 ± 3.4	19 ± 0.5
		− 1	6 ± 0.8	0.5 ± 0.02	4 ± 0.2	2 ± 0.1
pATSerInoG	2.7	+ 1	**89 ± 2.7**	53 ± 3.2	63 ± 3.2	30 ± 2.6
		− 1	11 ± 1	8 ± 2	9 ± 1	6 ± 0.5
pATSerUDAP	2.8	+ 1	46 ± 1.2	16 ± 0.47	37 ± 2.7	8 ± 0.55
		− 1	22 ± 1	7 ± 0.1	25 ± 1	6 ± 0.5
pATSerAG_GAAA_	3.1	+ 1	0.3 ± 0.01	0.4 ± 0.05	0.6 ± 0.05	0.07 ± 0.01
		− 1	6 ± 0.2	0.09 ± 0.01	7.3 ± 0.7	0.7 ± 0.1
pATSerCG_GAAA_	3.2	+ 1	0.8 ± 0.05	0.01 ± 0.002	5 ± 0.6	0.07 ± 0.004
		− 1	2.4 ± 0.2	0.05 ± 0.007	5 ± 0.5	0.8 ± 0.08
pATSerGG_GAAA_	3.3	+ 1	0.4 ± 0.06	0.46 ± 0.01	0.55 ± 0.1	0.04 ± 0.003
		− 1	7 ± 1	0.06 ± 0.001	5.5 ± 0.7	0.54 ± 0.04
pATSerUG_GAAA_	3.4	+ 1	22 ± 2	0.6 ± 0.05	13 ± 1	0.04 ± 0.006
		− 1	0.4 ± 0.005	0.03 ± 0.002	0.4 ± 0.01	0.01 ± 0.002
pATSerCA_GAAA_	3.5	+ 1	9 ± 0.8	0.14 ± 0.01	**26 ± 4**	1 ± 0.1
		− 1	0.1 ± 0.03	0.02 ± 1 × 10^–4^	ND	0.04 ± 0.002
pATSerUA_GAAA_	3.6	+ 1	10 ± 0.8	0.14 ± 0.01	3 ± 0.4	0.01 ± 0.002
		− 1	0.1 ± 0.01	0.02 ± 1 × 10^–4^	0.07 ± 0.01	0.01 ± 0.002

The data represent mean ± experimental errors calculated from at least three independent experiments and are expressed as cleavage per min per pmol of RPR. Dependent on RPR substrate combination, between 0.4 and 0.8 μM RPR was used, and 2 nM of substrate in all cases. The reactions were performed at 37 °C in buffer C at 800 mM Mg^2+^ (see “Materials and methods”) and the "substrate-N_248_" combinations showing the highest rates are highlighted in bold.

**Table 2:** Rate of cleavage (k_app_) of pMini3bp for RPR variants without the C5 protein.

	ExpS	CL site	A_248_ (wt)	RPR variant		
				C_248_	G_248_	U_248_
pMini3bpAG	4.1	+ 1	0.08 ± 0.001	0.001 ± 1 × 10^–4^	5.8 ± 0.6	0.044 ± 0.004
pMini3bpCG	4.2	+ 1	0.2 ± 0.007	0.002 ± 1 × 10^–4^	4 ± 0.3	0.02 ± 6 × 10^–4^
		− 1	0.03 ± 0.002	0.0001 ± 1 × 10^–5^	0.3 ± 0.002	0.005 ± 1 × 10^–4^
pMini3bpGG	4.3	+ 1	0.001 ± 7 × 10^–5^	0.0023 ± 2 × 10^–6^	0.003 ± 4.4 × 10^–4^	0.001 ± 3 × 10^–5^
		− 1	0.0006 ± 2 × 10^–5^	ND	0.007 ± 3 × 10^–4^	0.0008 ± 1 × 10^–5^
pMini3bpUG	4.4	+ 1	**16 ± 1**	0.02 ± 4.4 × 10^–4^	0.8 ± 6.4 × 10^–4^	0.02 ± 3.3 × 10^–5^
pMini3bpCA	4.5	+ 1	0.27 ± 0.07	0.012 ± 3.4 × 10^–4^	10 ± 1	0.22 ± 0.014
pMini3bpUA	4.6	+ 1	3.5 ± 1.2	0.028 ± 4.4 × 10^–4^	0.38 ± 0.044	0.003 ± 2.2 × 10^–4^
pMini3bpCIno	4.7	+ 1	1.1 ± 0.057	0.012 ± 0.0055	3.5 ± 0.099	0.12 ± 0.0075
pMini3bpDAPG	4.8	+ 1	0.026 ± 0.0031	0.00047 ± 1 × 10^–6^	1.5 ± 0.43	0.0052 ± 4.7 × 10^–4^
pMini3bpInoG	4.9	+ 1	0.027 ± 0.0023	0.035 ± 0.0033	0.017 ± 0.0033	0.0033 ± 6 × 10^–4^
pMini3bpPuG	4.10	+ 1	0.0075 ± 2.5 × 10^–4^	0.0012 ± 1.7 × 10^–5^	0.095 ± 0.0014	0.0093 ± 0.0014
pMini3bpUDAP	4.11	+ 1	0.52 ± 0.064	0.0077 ± 0.0019	0.42 ± 0.0016	0.0079 ± 0.0019
pMini3bp2APG	4.12	+ 1	0.0088 ± 5.5 × 10^–4^	0.00076 ± 3 × 10^–5^	0.036 ± 0.0014	0.0046 ± 7.7 × 10^–4^

The data represent mean ± experimental errors calculated from at least three independent experiments and are expressed as cleavage per min per pmol of RPR. Dependent on RPR substrate combination, between 0.4 and 0.8 μM RPR was used, and 2 nM of substrate in all cases. The reactions were performed at 37 °C in buffer C at 800 mM Mg^2+^ (see “Materials and methods”) and the "pMini3bp-N_248_" combination showing the highest rate is highlighted in bold.

**Table 3:** Rate of cleavage (k_app_) of as a function of having 3-methyl U at −1.

	ExpS	CL site	A_248_ (wt)	RPR variant		
				C_248_	G_248_	U_248_
pATSerUG	2.4	+ 1	73 ± 2	32 ± 4	61 ± 0.4	11 ± 0.2
pATSer**3m**UG	**5.1**	+ 1	18 ± 2	8 ± 1	15 ± 0.2	4 ± 0.6
pATSerUG_GAAA_	3.4	+ 1	22 ± 2	0.6 ± 0.05	13 ± 1	0.04 ± 0.006
		− 1	0.4 ± 0.005	0.03 ± 0.002	0.4 ± 0.01	0.01 ± 0.002
pATSer**3m**UG_GAAA_	**5.2**	+ 1	6 ± 0.3	0.4 ± 0.05	2 ± 0.1	0.05 ± 0.002
		− 1	0.2 ± 0.02	0.04 ± 0.005	0.1 ± 0.01	ND
pMini3bpUG	4.4	+ 1	16 ± 1	0.02 ± 4.4 × 10^–4^	0.8 ± 6 × 10^–4^	0.02 ± 3.3 × 10^–5^
pMini3bp**3m**UG	**5.3**	+ 1	1 ± 0.001	0.05 ± 0.001	0.3 ± 0.01	0.02 ± 0.001

The data represent mean ± experimental errors calculated from at least three independent experiments and are expressed as cleavage per min per pmol of RPR. Dependent on RPR substrate combination, between 0.4 and 0.8 μM RPR was used, and 2 nM of substrate in all cases. The reactions were performed at 37 °C in buffer C at 800 mM Mg^2+^ (see “Materials and methods”). The bold ExpS numbers highlight the substrates with **3mU**.

Following this, in “Kinetic constants k_obs_ and k_obs_/K^sto^ and activation energy as a function of N_248_ identity” we present single turnover kinetic data for cleavage of the model substrate pATSerUG with the different 248 variants. These experiments were performed at different temperatures with the objective to determine the activation energy as a function of the N_248_ identity. In the final “Differential effects due to replacement of the 2ʹOH at −1 with 2ʹH or 2ʹNH_2_ in pATSerUG and influence of the N_248_ identity on the charge distribution at the cleavage site”, we probe the influence of the N_248_ identity on the charge distribution at the cleavage site.

##### Substrates that can interact productively with the TBS-region—influence of changes of the nucleobase at −1 in substrates and residue 248 in the RPR

*pSu1 variants* (Fig. 4A and Table 1; ExpS 1.1–1.4): All the −1 variants were cleaved mainly at the +1 site and at the alternative position −1 (Fig. 4A), irrespective of the identity of residue 248. Except for U_248_, the U_−1_ variant (ExpS 1.4) was cleaved with the highest rate at +1 where k_app(+1)_ was highest for A_248(wt)_. With respect to the wild-type substrate, pSu1C_−1_ (ExpS 1.2), A_248(wt)_ was the most efficient catalyst; the k_app_ values for the other three variants were lower.

For pSu1A_−1_ (ExpS 1.1), changing *Eco* RPR_A248(wt)_ to any of the other nucleobases resulted in decreased cleavage frequency at +1 relative to −1, while comparing k_app_ values for cleavage at +1 and −1 with the different RPRs differed ≤ two-fold. Cleavage of pSu1C_−1_ (ExpS 1.2) at +1 was reduced and increased at −1 using G_248_, but there was no apparent difference for the other 248 variants. The increased cleavage at −1 for G_248_ is also noticeable by comparing k_app(+1)_ and k_app(−1)_ values (Table 1). The wild-type A_248_ and G_248_ variant cleaved pSu1G_−1_ at +1 with slightly lower frequencies than C_248_ and U_248_ (ExpS 1.3). Moreover, k_app(+1)_ values for all 248 variants were similar (ExpS 1.3) while k_app_ for cleavage at −1 were higher for A_248(wt)_ and G_248_ compared to C_248_ and U_248_. Finally, with pSu1U_−1_ (ExpS 1.4) we detected modest differences in cleavage frequency at +1 and k_app(+1)_ (at most 2.6-fold change comparing A_248(wt)_ and U_248_) while no apparent change in k_app(−1)_ was detected irrespective of RPR. We also noted that the 248 variants cleaved the different pSu1 N_−1_ substrates with low frequencies at other positions in the 5ʹ leader upstream of site −1 (not shown). Noteworthy, in *E. coli* the wild-type pSu1 has a C at position −1, which pairs with the discriminator base G_+73_ (Fig. 3A) and the C_−1_/G_+73_ pairing influences cleavage efficiency and site selection, see e.g.^27^.

*pATSer variants* (Fig. 4B and Table 1; ExpS 2.1–2.8): Cleavage of the N_−1_ variants carrying A, C, G and U more or less mirrored the results with pSu1 (cf. ExpS 1.1–1.4 vs. 2.1–2.4). Overall G_248_ cleaved with the highest rates both at +1 (pATSerAG; ExpS 2.1) and at −1 (pATSerGG; ExpS 2.3). Moreover, having a purine at 248 gives a more efficient catalyst compared to when a pyrimidine is present at this position with the exception of the "pATSerCG/G_248_" combination (ExpS 2.2).

Specifically, first pATSerAG (ExpS 2.1) was cleaved with roughly the same frequencies at +1 by all 248 variants with the possible exception for C_248_, which cleaved this substrate both at +1 and −1 with a lower rate compared to the other RPR variants. Second, compared to the other RPRs G_248_ cleaved pATSerCG more frequently at −1. This is also reflected in the k_app_ values for cleavage at +1 and −1, while the G_248_ and C_248_ RPRs cleaved pATSerCG at +1 with the same rates (ExpS 2.2). These results are contradictory to the formation of *cis* WC/WC pairing between C_−1_ and G_248_ in the RPR-substrate complex. Third, in contrast there appeared to be suppression/rescue of cleavage of pATSerGG at −1 using C_248_ (comparing frequencies of cleavage at −1 and k_app(−1)_; Fig. 4B and Table 1; cf. ExpS 2.3 C_248_ vs. G_248_). Fourth, pATSerUG (cf. ExpS 2.4) was almost exclusively cleaved at +1 by all 248 variants (see also Fig. 3F). However, U_248_ cleaved pATSerUG with a significantly lower rate at +1 than the other RPR variants (Table 1).

For the pATSer variants with "unnatural" nucleobases at −1 and +73, reducing the number of potential hydrogen bonds between −1 and +73 from three to two restored cleavage at +1 for G_248_ to the level observed for the other 248 variants (Fig. 4B; cf. ExpS 2.2 and 2.5, i.e. pATSerCG vs. pATSerCIno). This appeared to be the result of an increase in the rate of cleavage at +1 while hardly any effect on the rate was detected for cleavage at −1 (Table 1; cf. ExpS 2.2 and 2.5). By contrast, the potential formation of three hydrogen bonds between U_−1_ and DAP_+73_ resulted in increased miscleavage for all four 248 variants (Fig. 4B; cf. ExpS 2.4 and 2.8, i.e. pATSerUG vs. pATSerUDAP). This was accompanied with noticeable rates of cleavage at −1 (Table 1; cf. ExpS 2.4 and 2.8). In keeping with this, the k_app_ values for cleavage at +1 were lower for pATSerUDAP relative to pATSerUG for all RPR variants.

**Figure 5:**
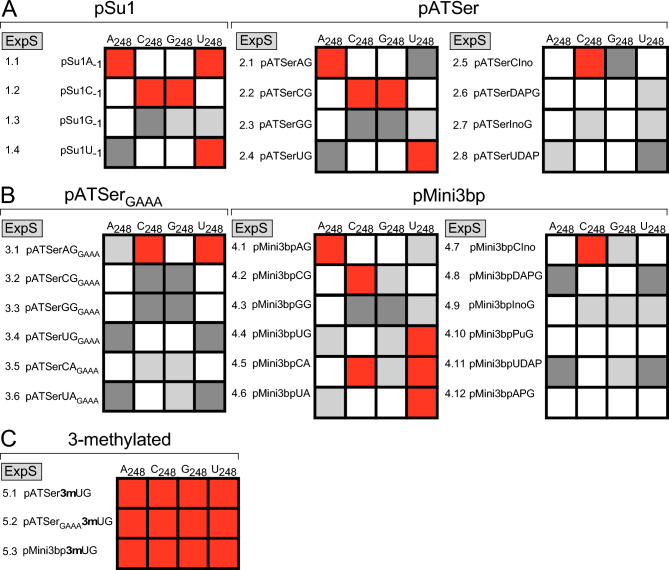
Summary of data for N_−1_/N_248_ *cis* WC/WC base paring. Boxes marked in gray are consistent with *cis* WC/WC base-pairing; light gray marks those combinations where one combination (or weak agreement/non-WC/WC pairing e.g. GU-pairing) are consistent with *cis* WC/WC base-pairing, e.g. cf. pSu1U_−1_/A_248_- vs pSu1A_−1_/U_248_-combinations. Boxes marked in red highlight the combinations that are not in agreement with *cis* WC/WC base pairing, while no color indicates other combinations. The grey ExpS boxes refer to the Experimental Series, e.g. 1.1–1.4 and 2.1–2.8 etc., as shown in Figs. 4 and 6, and Tables 1, 2 and 3. (**A**) Experiment series using pSu1 (ExpS 1.1–1.4) and pATSer (ExpS 2.1–2.8) variants, which can establish a productive interaction with the TBS region in the S-domain (see main text for details). (**B**) Experiment series using pATSerGAAA (ExpS 3.1–3.6) and pMini3bp (ExpS 4.1–4.12) variants, which cannot form a productive interaction with the TBS region in the S-domain (see main text for details). (**C**) Experiment series for model substrates with a 3-methyl group at U_−1_ (ExpS 5.1–5.3).

In summary (see Fig. 5A), (i) when the T-loop can form a productive interaction with TBS in the S-domain we did not detect any conclusive evidence for *cis* WC/WC pairing between N_−1_ and N_248_. However, for some of the combinations, *cis* WC/WC pairing cannot be excluded (see also the “Discussion”). (ii) The potential to form three H-bonds between N_−1_ and N_+73_ affected both cleavage site selection and dependent on substrate-RPR combination the rate of cleavage. (iii) For *Eco* RPR_A248(wt)_, cleavage of substrates with natural nucleobases at N_−1_, the U_−1_ substrates are preferred.

**Figure 6:**
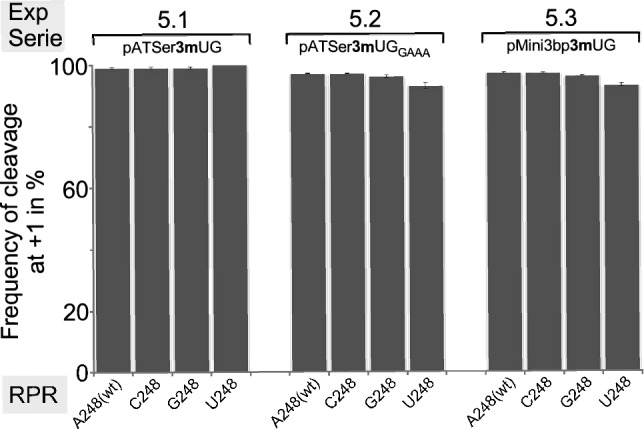
Frequencies of cleavage-site selection for 3-methylated substrates by *Eco* RPR 248 variants. Histograms summarizing frequencies of cleavage at +1 in % during *Eco* RPR-mediated cleavage of pATSer**3m**UG (ExpS 5.1), pATSer**3m**UG_GAAA_ (ExpS 5.2) and pMini3bp**3m**UG (ExpS 5.3) as indicated. We used the 5ʹ cleavage fragments to calculate the frequencies of cleavage at +1; mean and experimental errors were calculated from at least three independent experiments.

##### Substrates that cannot interact productively with the TBS-region—influence of changes of the nucleobase at −1 in substrates and residue 248 in the RPR

*pATSer GAAA-tetra loop variants* (Fig. 4C and Table 1; ExpS 3.1–3.6): Replacement of the T-loop with a GAAA-tetra loop in the pATSer variants resulted in reduced frequency of cleavage at +1 for several combinations and lower k_app(+1)_ (cf. ExpS 2.1–2.4 vs. 3.1–3.4). However, dependent on substrate-RPR combination the decrease in rate at +1 varied between ≈ two- to 1000-fold; for example the k_app(+1)_ ratio for pATSerCG/pATSerCG_GAAA_ and C_248_ showed a 1000-fold difference, but only ≈two-fold for pATSerCG/pATSerCG_GAAA_ and G_248_ (Table 1; ExpS 2.2 vs. 3.2, cf. k_app_ 10 vs. 0.01 and 11 vs. 5, respectively). For cleavage at −1, the decrease in k_app(−1)_ was lower (≤ 20-fold) in response to substituting the T-loop with GAAA for all four 248 variants except for cleavage of pATSerGG vs. pATSerGG_GAAA_ with C_248_. Here the decrease in (k_app(−1)_) was > 100-fold (Table 1; ExpS 2.3 vs. 3.3). Moreover, for the "pATSerAG_GAAA_/C_248_", "pATSerCG_GAAA_/G_248_" and "pATSerGG_GAAA_/C_248_" combinations, we did observe significant cleavage at +1 with frequencies (relative to −1) comparable to those with an intact T-loop (e.g. cf. C_248_ cleavage of pATSerAG and pATSerAG_GAAA_; Fig. 4B,C; ExpS 2.1 vs. 4.1). Substitution of the T-loop with the GAAA-tetra loop in pATSerUG increased cleavage at −1 for U_248_, while we detected only small changes for the other 248 variants including the wild type (cf. Fig. 4B,C; ExpS 2.4 vs. 3.4). However, all RPR variants cleaved pATSerUG_GAAA_ with measurable k_app(−1)_ values in contrast to pATSerUG (Table 1; ExpS 2.4 vs. 3.4). In keeping with the importance of pairing between N_−1_ and N_+73_ (see above) changing G to A at +73 in pATSerCG_GAAA_ restored cleavage at +1 with increased rates irrespective of N_248_ variants (Fig. 4C and Table 1; cf. pATSerCG_GAAA_ vs. pATSerCA_GAAA_, ExpS 3.2 and 3.5). By contrast, comparing k_app(+1)_ values for cleavage of pATSerUG_GAAA_ and pATSerUA_GAAA_ showed the opposite, i.e., lower k_app(+1)_ irrespective of 248 variant (Table 1; ExpS 3.4 vs. 3.6). For pATSerUA_GAAA_, increased frequency of cleavage at −1 was detected with U_248_ and it also cleaved pATSerUA_GAAA_ at position −2 (≈20%) in the 5ʹ leader. Finally, a comparison of k_app(+1)_ values (and to some extent also k_app(−1)_) suggested that A_248(wt)_ and G_248_ were more efficient catalysts than C_248_ and U_248_ when pATSerNN_GAAA_ variants were used.

*pMini3bp variants* (Fig. 4D and Table 2; ExpS 4.1–4.12): For the variants with natural nucleobases at −1 we did observe significant reduction in cleavage of the G_−1_ variant at +1 using A_248(wt)_, G_248_ and U_248_ while C_248_ cleaved the G_−1_ substrate preferentially at +1 (Fig. 4D; ExpS 4.3). Moreover, irrespective of 248 variants pMini3bpCG and pMini3bpCA were cleaved mainly at +1 with some cleavage at −1 and cleavage of pMini3bpUA was detected only at +1 (Fig. 4D; ExpS 4.2, 4.3 and 4.5). The other three variants, pMini3bpAG, pMini3bpUG and pMini3bpUA (ExpS 4.1, 4.4 and 4.6), were cleaved almost exclusively at +1 by all four 248 variants.

With respect to the rate of cleavage we compared k_app(+1)_ (except for two substrates, see below) because the rates were significantly lower than for the other substrates, in particular at −1 (Table 2). Overall, k_app(+1)_ for A_248(wt)_ and G_248_ were higher compared to C_248_ and U_248_; the highest k_app(+1)_ was for pMini3bpUG (ExpS 4.4) with A_248(wt)_. This is in keeping with the trend seen with the other substrate variants, i.e. U_−1_ variants were in general cleaved with the highest rates at +1 (but cf. e.g., the "pATSerAG/G_248_" combination above). For the "pMini3bpCG/G_248_" combination (ExpS 4.2), k_app(+1)_ and k_app(−1)_ were both higher than when the other 248 variants, including A_248(wt)_, were used. Comparing cleavage of pMini3bpCG vs. pMini3bpGG (ExpS 4.2 and 4.3) with G_248_, k_app(+1)_ was ≈1300-fold higher for pMini3bpCG, while k_app(−1)_ was ≈40-fold higher. No difference in k_app(+1)_ was detected for C_248_ cleaving these two substrates, while k_app(−1)_ was 3000-fold lower in cleaving pMini3bpCG than when G_248_ was used (Table 2; ExpS 4.2 and 4.3, cf. 0.0001 vs. 0.3). In fact, C_248_ was found to be a very poor catalyst with all pMini3bp substrates. Analyzing the (pMini3bpUG and pMini3bpAG)/A_248(wt)_ and (pMini3bpUG and pMini3bpAG)/U_248_ combinations (ExpS 4.1 and 4.4) revealed a significant drop in k_app(+1)_ for A_248(wt)_ by replacing U_−1_ with A_−1_, but only a two-fold rescue (cf. pMini3bpUG vs. pMini3bpAG) using U_248_. Interestingly, G_248_ cleaved the pMini3bpAG substrate at +1 with a markedly higher rate (k_app(+1)_) compared to using the other 248 variants (Table 2; ExpS 4.1).

For the variants carrying "unnatural" nucleobases at −1 and/or at +73, most combinations resulted in cleavage mainly at +1 (Fig. 4D). The most apparent exceptions were for the combinations "pMini3bpDAPG/A_248(wt)_", "pMini3bp2APG/A_248(wt)_", and "pMini3bpUDAP/U_248_".

The pMini3bp variants cannot interact with the TBS-region (see above) and comparison of the pATSer (with T-loop) and the pMini3bp data sets (cf. Fig. 4B,D) revealed that some pATSer variants such as pATSerAG (ExpS 2.1), pATSerInoG (ExpS 2.7) and pATSerUDAP (ExpS 2.8; except U_248_) were cleaved at −1 with higher frequencies by all four 248 variants. Relative to cleavage of the pATSer derivatives with GAAA-tetra loops, the frequencies of cleavage at +1 were, in general, higher with the pMini3bp variants.

Considering rates of cleavage, introduction of 2NH_2_ [Table 2; cf. pMini3bpAG (ExpS 4.1) vs. pMini3bpDAPG (ExpS 4.8)] and removal of the 6NH_2_ [Table 2; cf. pMini3bpAG (ExpS 4.1) vs. pMini3bp2APG (ExpS 4.12)] on the −1 nucleobase resulted in a ≈four- and ≈ 160-fold decrease in k_app(+1)_ for G_248_, while for A_248(wt)_ the corresponding values were ≈ three- and ≈ ten-fold lower. These data suggested that in particular the exocyclic amine at position 6 on A_−1_ plays a more important role for cleavage with G_248_ than for A_248(wt)_. Cleavage of pMini3bpUG and pMini3bpUA with G_248_ resulted in a 20- and ten-fold lower k_app(+1)_, respectively, compared to A_248(wt)_ (Table 2; ExpS 4.4 and 4.6) while only a small difference in k_app(+1)_ was detected for cleavage of pMini3bpUDAP using these two RPRs (Table 2; ExpS 4.11). Moreover, the k_app(+1)_ values for these three pMini3bp U_−1_ substrates using G_248_ were similar, within a factor of two. This might indicate that the catalytic performance of A_248(wt)_ is influenced by pairing between N_−1_ and N_+73_ and/or the pairing between N_+73_ and U_294_ in the RPR-substrate complex.

In summary (see Fig. 5B), (i) the cleavage site distribution data did not provide any conclusive evidence for *cis* WC/WC pairing between residues N_−1_ and 248 in the *Eco* RPR substrate complex when we interfered with/or removed the interaction between the T-loop and TBS. However, there were a few possible exceptions, e.g., the combinations "pATSerCG_GAAA_/G_248_", "pATSerGG_GAAA_/C_248_", and "pMini3bpGG/C_248_". (ii) As in cleavage of pSu1 and pATSer variants, the potential pairing between N_−1_ and N_+73_ influence the efficiency of cleavage and site selection also in the absence of a productive interaction between the T-loop and TBS in the RPR. (iii) Interfering with the interaction between TSL and TBS affect choice of cleavage site and rate of cleavage, see also^15,37^.

Together the combined data with the four different substrate series suggested that the influence of N_−1_ and N_+73_ on cleavage site recognition and rate of cleavage at +1 and −1 depend on substrate and/or "N_−1_/N_+73_-N_248_" combination. Moreover, in general we do detect larger variations in k_app_ for cleavage at +1 than at −1. It therefore appears that the impact of the various changes either in the substrate or in the RPR is larger for cleavage at the correct position +1 than at −1. We also emphasize that the choice of cleavage site did not change during the course of the reactions as revealed from the time course experiments used to determine k_app_ values.

##### Altering the WC-surface of a U at position −1 and influence of the N_248_ identity

To further understand the importance of the Watson–Crick surface of the N_−1_ residue in the substrate we used substrates carrying substitutions of U with 3-methyl U (**3mU**) at −1. This modification would be expected to disturb the interaction with the Watson–Crick surface of U_−1_ (Fig. 3B–E). The data are shown in Fig. 6 and Table 3 (ExpS 5.1–5.3 vs. 2.4, 3.4 and 4.4).

A comparison of cleavage of pATSerUG vs. pATSer**3m**UG revealed no (or very minor) change in choice of cleavage site (Figs. 4B and 6; cf. ExpS 2.4 vs. ExpS 5.1) for any of the four 248 variants. However, k_app(+1)_ dropped three- to four-fold for all four RPRs, with U_248_ being the least efficient catalyst (Table 3).

With pATSerUG_GAAA_, introduction of **3mU** at −1 did not result in any apparent change in cleavage site preference with the notable exception for U_248_. Here we did detect an increase of cleavage at +1 compared to cleavage of pATSerUG_GAAA_ (Figs. 4C and 6; cf. ExpS 3.6 vs. ExpS 5.2). The other three 248 variants cleaved both pATSerUG_GAAA_ and pATSer**3m**UG_GAAA_ at +1. As for pATSerUG, the presence of **3mU**_−1_ influenced cleavage rates; k_app(+1)_ values were down four- to six-fold using A_248(wt)_ and G_248_, respectively, while for C_248_ the decrease was very modest, ≈1.5-fold. No apparent change was detected for U_248_. Interestingly, **3mU**_−1_ influenced the rate of cleavage at +1 for A_248(wt)_ and C_248_ while cleavage by G_248_ resulted in a four-fold decrease (Table 3; cf. ExpS 3.4 vs. ExpS 5.2).

Comparing cleavage of pMini3bpUG vs. pMini3bp**3m**UG, we detected just a small increase in cleavage at −1 for all 248 variants (Figs. 4D and 6; cf. ExpS 4.4 vs. ExpS 5.3). Moreover, k_app(+1)_ for A_248(wt)_ was down 16-fold in response to the introduction of **3mU**_−1_. For G_248_ and C_248_, the change was more modest, 2.7-fold lower for G_248_ while C_248_ cleaved **3mU**_−1_ with a 2.5-fold higher rate than it cleaved the corresponding substrate lacking the methyl modification. No change was detected for U_248_.

In summary (see Fig. 5C), the presence of **3mU**_−1_ that blocks the Watson–Crick surface has an impact on the rate of cleavage. The impact on the rate at +1 (k_app(+1)_) appears to be dependent on RPR-substrate combination, as exemplified by cleavage of pMini3bpUG and pMini3bp**3m**UG with A_248(wt)_ vs. C_248_. Remarkably, introduction of **3mU**_−1_ in the "pATSer-GAAA-tetra-loop" substrate rescued cleavage at +1 using the U_248_ RPR variant. Hence, these findings do not support *cis* WC/WC pairing between N_−1_ and 248 for these substrates, see also^29^.

##### Kinetic constants k_obs_ and k_obs_/K^sto^ and activation energy as a function of N_248_ identity

**Figure 7:**
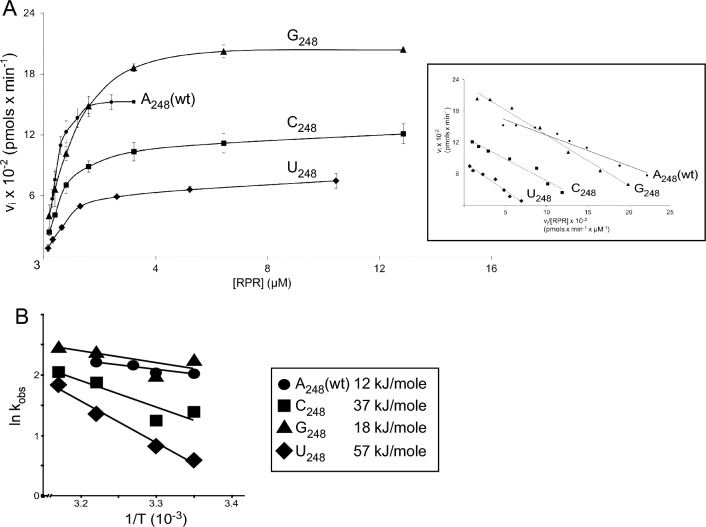
Kinetics of cleavage of pATSer with the *Eco* RPR 248 variants and Arrhenius plots. (**A**) Rate of cleavage of pATSerUG as a function of increasing concentration of the *Eco* RPR 248 variants. The experiments were performed at 37 °C in buffer C containing 800 mM Mg^2+^ as described in “Materials and methods”. The data represent mean and experimental errors from at least three independent experiments. Insets correspond to Eadie–Hofstee plots using the primary data and the k_obs_ and k_obs_/K^sto^ values presented in Table 4. (**B**) Arrhenius plots of temperature dependence of k_obs_ for the *Eco* RPR_248_ variants as indicated. The data are summarized in Table 4 and the temperatures are in Kelvin. The values given in the inset correspond to the calculated E_a_ (activation energy) values.

The data presented above clearly suggested that the identity of residue 248 affect both cleavage site recognition and rate of cleavage. We therefore decided to determine the kinetic constants, k_obs_ and k_obs_/K^sto^ (for cleavage at +1), for the different 248 variants using pATSerUG. To gain insight into why a purine at 248 (in particular A at 248) is preferable over a pyrimidine, we also determined k_obs_ and k_obs_/K^sto^ at different temperatures. This would allow us to estimate the activation energy for the reaction catalyzed by the various 248 RPRs. These series of experiments were done under single turnover conditions at 800 mM Mg^2+^ (see above) and the results are shown in Fig. 7 and Table 4.

**Table 4:** The kinetic constants for cleavage of pATSerUG at as a function of temperature and 248 variant.

248 variant	Temp (°C)	k_obs_ (min^-1^)	k_obs_/K^sto^ (min^-1^ μM^-1^)	K^sto^ ≈K_d_ (μM)	E_a_ (kJ mol^-1^)
A_248_	25	7.6 ± 0.32	9.3 ± 2.6	0.9 ± 0.29	12
	30	7.7 ± 0.5	11 ± 1	0.7 ± 0.024	
	33	8.6 ± 0.27	7.2 ± 0.44	1.2 ± 0.039	
	37	9.1 ± 0.035	16 ± 3.4	0.6 ± 0.12	
C_248_	25	4.0 ± 0.37	5.2 ± 1.6	0.92 ± 0.42	37
	30	3.5 ± 0.25	7.4 ± 1.8	0.51 ± 0.14	
	37	6.5 ± 0.10	7.9 ± 0.63	0.84 ± 0.066	
	42	7.8 ± 0.41	14 ± 1.3	0.58 ± 0.069	
G_248_	25	9.4 ± 0.24	16 ± 2	0.6 ± 0.094	18
	30	7.4 ± 0.49	8.3 ± 3.5	1.3 ± 0.75	
	37	11 ± 0.44	13 ± 3.6	1 ± 0.27	
	42	12 ± 0.18	16 ± 1.5	0.78 ± 0.078	
U_248_	25	1.8 ± 0.066	3.2 ± 0.4	0.55 ± 0.054	57
	30	2.3 ± 0.21	6.1 ± 0.36	0.37 ± 0.057	
	37	3.9 ± 0.21	3.6 ± 0.42	1.1 ± 0.16	
	42	6.3 ± 0.18	6 ± 0.73	1.1 ± 0.18	

The experiments were performed under single-turnover conditions at 800 mM Mg^2+^ concentrations at pH 6.1 as outlined in “Materials and methods”. For details regarding the calculation of K_d_, see the main text, Wu et al.^31^ and references therein. The activation energies were calculated using the k_obs_ values as described in Tallsjö and Kirsebom^68^ (see also Fig. 7). The data represent mean ± experimental errors calculated from at least three independent experiments.

The k_obs_ and k_obs_/K^sto^ values for A_248(wt)_ at 37 °C agreed with our previous data (Table 4)^37^. A comparison of k_obs_ and k_obs_/K^sto^ for the four 248 variants revealed that having A or G at 248 resulted in the most efficient catalysts, in agreement with data discussed above. For A_248(wt)_, lower temperature resulted in a modest but reproducible decrease in k_obs_. This trend was also detected for the other 248 variants. The k_obs_ at different temperatures were highest for A_248(wt)_ and G_248_, and lowest for C_248_ and U_248_. Irrespective of 248 variant and temperature, the K^sto^ values were similar within a ≈ two- to three-fold range. We have argued that under these reaction conditions K^sto^ ≈ K_d_ (see “Materials and methods”)^31^ and references therein. On the basis of this, our data suggested that substituting A_248(wt)_ resulted in a modest change in binding affinity for pATSerUG.

Notwithstanding that the variation in k_obs_ in response to temperature was modest (but reproducible) we plotted k_obs_ as a function of temperature (Arrhenius plot). This would give an indication about the activation energy (E_a_) for cleavage of pATSerUG by the different 248 variants. The E_a_ values varied from 12 to 57 kJ/mole, with A_248(wt)_ having the lowest value followed by G_248_ < C_248_ and < U_248_ (Fig. 7; Table 4).

Taken together, in keeping with the data discussed above, a purine at 248 is preferred over a pyrimidine, with U_248_ being the weakest catalyst. From these data it also appears that this is, at least in part, due to the activation energy barrier being lower with a purine at 248, in particular with an adenosine as in *Eco* RPR_A248(wt)_. This provides one rational why A at position 248 in bacterial RPR (*Eco* numbering) is conserved (see also the “Discussion”).

##### Differential effects due to replacement of the 2ʹOH at −1 with 2ʹH or 2ʹNH_2_ in pATSerUG and influence of the N_248_ identity on the charge distribution at the cleavage site

The 2ʹOH of residue −1 is important for both cleavage rates and site selection in bacterial RPR-mediated catalysis^43^. Hence, we decided to investigate whether replacement of the U_−1_ 2ʹOH with 2ʹH or 2ʹNH_2_ in pATSerUG (pATSer**d**UG and pATSer**am**UG, respectively; Fig. 3B,E) influenced the choice of cleavage site.

**Figure 8:**
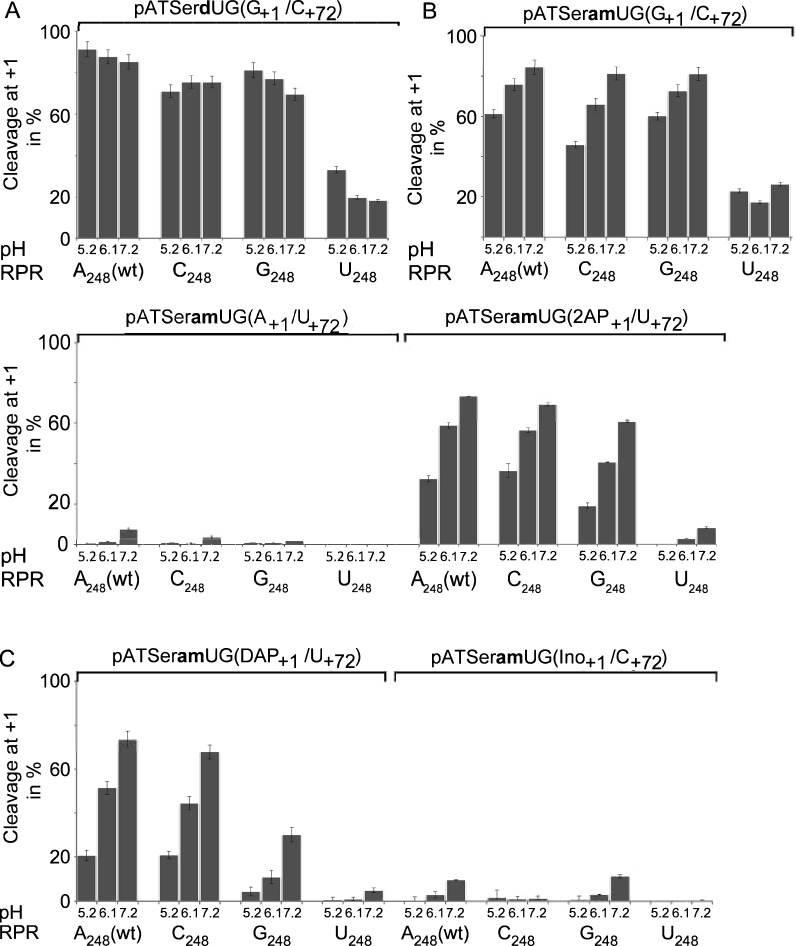
Frequencies of cleavage at +1 of different pATSerUG derivatives with 2ʹH or 2ʹNH_2_ at the −1 position at different pHs by *Eco* RPR 248 variants. (**A**) Histograms summarizing frequencies of cleavage at +1 in % during *Eco* RPR-mediated cleavage of pATSer**d**UG (2ʹOH at −1 substituted with 2ʹH). (**B**) Histograms summarizing frequencies of cleavage at +1 in % during *Eco* RPR-mediated cleavage of pATSer**am**UG (2ʹOH at −1 substituted with 2ʹNH_2_) and pATSer**am**UG(2AP_+1_/U_+73_). (**C**) Histograms summarizing frequencies of cleavage at +1 in % during *Eco* RPR-mediated cleavage of pATSer**am**UG(A_+1_/U_+73_) and pATSer**am**UG(2AP_+1_/U_+73_). (**D**) Histograms summarizing frequencies of cleavage at +1 in % during *Eco* RPR-mediated cleavage of pATSer**am**UG(DAP_+1_/U_+73_) and pATSer**am**UG(Ino_+1_/C_+73_). We used the 5ʹ cleavage fragments to calculate the frequencies of cleavage at +1 at different pH as indicated; mean and experimental errors were calculated from at least three independent experiments. For experimental details see “Materials and methods”.

Introduction of a 2ʹH (pATSer**d**UG) resulted in reduced cleavage at +1 for all 248 variants irrespective of pH (5.2, 6.1 and 7.2) consistent with previous data using pre-tRNA^24,25^. Importantly, cleavage at −1 did not increase with pH (Fig. 8A). Cleavage of the 2ʹNH_2_ substituted substrate (pATSer**am**UG) on the other hand resulted in increased cleavage at +1 at higher pH. In contrast to cleavage with A_248(wt)_ and G_248_ higher pH was required to reach 50% cleavage at +1 using C_248_ (Fig. 8B). The most dramatic effect however, was observed using the U_248_ variant. Here we did not detect any significant change in the frequency of cleavage at +1 with increasing pH.

The pH dependent cleavage of pATSer**am**UG at +1 by *Eco* RPR_A248(wt)_ is also influenced by the identity of N_+1_/N_+72_ (cf. Fig. 5 in^40^; see also^44^; Fig. 8B,C; cf. G_+1_/C_+72_, A_+1_/U_+72_, 2AP_+1_/U_+72_, DAP_+1_/U_+72_ and Ino_+1_/C_+72_ substrate variants). This was also the case for the C_248_ and G_248_ variants. Of those substrate variants having an exocyclic amine at position 2 on the nucleobases (2NH_2_) at +1 (Fig. 3B; cf. substrates with G_+1_, 2AP_+1_ and DAP_+1_) C_248_ showed a similar response to pH as A_248(wt)_, while higher pH was needed to reach 50% cleavage at +1 for G_248_ except using pATSer**am**UG(G_+1_/C_+72_) (cf. Fig. 8A–C). For the substrates lacking a 2NH_2_ on the nucleobase at +1 [pATSer**am**UG(A_+1_/U_+72_) and pATSer**am**UG(Ino_+1_/C_+72_)], we detected only a small increase in cleavage at +1 with increasing pH for A_248(wt)_, C_248_ and G_248_ while for U_248_ no cleavage at +1 was observed. In fact, for U_248_ we observed no or only a small increase in cleavage at +1 using all pATSer**am**UG(N_+1_/N_+72_) variants with increasing pH. For all the RPR substrate combinations we also detected cleavage at other positions both downstream of the +1 site and in the 5ʹ leader with increasing pH (not shown). Also, irrespective of residue at 248 no significant change in the frequencies of cleavage at +1 with changing pH using the all ribo substrate variants was detected (not shown).

Taken together, these data suggest that the protonation (the pKa value) of the 2ʹNH_2_ at −1 is affected by the nucleobase identity at position 248 in *Eco* RPR and at +1 (and +72) in pATSerUG (see “Discussion”).

## Discussion

Residues in the RNase P substrate interact with several regions of the RNA subunit (RPR) of bacterial RNase P (see introduction). Among these the N_−1_ residue in the substrate 5ʹ leader is close to the active center where cleavage occurs, and it has been proposed that the well conserved A_248(wt)_ forms a *cis* WC/WC base pair when U is present at −1^24,25^. These studies were primarily based on using pre-tRNAs carrying different deoxyribonucleobases at position N_−1_. In *E. coli* ≈40% of the pre-tRNAs do not carry a U at −1^24,27,28^. Also, cross-linking studies suggest that N_−1_ and N_+1_ in the substrate are positioned close to A_248_-C_253_ and G_332_-A_333_ (*E. coli* numbering, see Fig. 1A)^26,45,46^. Hence, we have argued that A_248(wt)_ is a key nucleobase of a N_−1_ binding surface/pocket^16,27,29^. Here we provide data where we analyzed cleavage as a function of A_248(wt)_ substitutions and N_−1_ nucleobase identity using all ribo pre-tRNA and three all ribo model substrates to investigate whether N_−1_ and N_248_ forms a *cis* WC/WC base pair. If *cis* WC/WC base pair forms between N_−1_ and N_248_ this means that the phenotypic change due to disruption of the N_−1_/N_248_ pairing can be rescued by a compensatory change that restores pairing between N_−1_/N_248_. For the pre-tRNA substrate pSu1 and the model substrate pATSer, which both can form a productive TSL/TBS-interaction (see “Introduction”, induced fit mechanism)^15,30,37,46^, the data supported *cis* WC/WC pairing for substrates carrying G at −1, while we did not find any conclusive evidence for *cis* WC/WC pairing using the other combinations (except the U_−1_/A_248_ vs. A_−1_/U_248_ combinations in the pATSer context; see summary, Fig. 5A). When we interfered with the TSL/TBS-interaction by using "pATSer-GAAA-tetra-loop" substrates our findings are consistent with *cis* WC/WC pairing using the C_−1_, G_−1_ and U_−1_ substrate variants but not for A_−1_ (see summary, Fig. 5B). The impact of the N_−1_/N_248_ interaction was also detected using pre-tRNA substrates carrying a 2ʹH at −1 or substrates that could not form the RCCA-RNase P RNA interaction^24,25^, i.e. when additional RPR substrate interactions were disrupted. Moreover, our findings with the pMini3bp variants, which cannot interact with TBS in the S domain, lend less support for *cis* WC/WC pairing than when the "pATSer-GAAA-tetra-loop" series was used. But, support comes from using pMini3bpGG, pMini3bpDAPG and pMini3bpUDAP, where the latter can form three hydrogen bonds between N_−1_ and N_+73_ in the substrate (see summary, Fig. 5B). In summary, detection of possible *cis* WC/WC pairing between N_−1_ and N_248_ depends on substrate and disruption of more than one RPR-substrate contact such as the TSL/TBS-interaction.

Residue A_248_ is well conserved among bacterial RPRs and if the U_−1_ WC surface are involved in pairing with residue A_248(wt)_ blocking the N3 position on the nucleobase—by adding a methyl group (**3mU**)—would interfere with choice of cleavage site and rate of cleavage. As in pSu1, the model substrates carry an A at −2. Hence, following Zahler et al.^24,25^, who used pre-tRNA^Asp^ that also carries A_−2_, we argued that interfering with the formation of the "U_−1_/A_248(wt)_" potential pairing would result in a shift of cleavage from the correct site to the alternative site −1 due to the presence of the 3-methyl group at the N3 position of U_−1_ in the substrate. All three **3mU**_−1_ model substrate variants were, however, preferentially cleaved at +1 irrespective of 248-variant. This is inconsistent with *cis* WC/WC pairing (see summary, Fig. 5C). Importantly, the introduction of **3mU**_−1_ in the three all ribo model hairpin loop substrates did not shift choice of cleavage site for wild type *Eco* RPR_A248(wt)_, which would be expected if there was *cis* WC/WC pairing between U_−1_ and A_248(wt)_, see also^29^. It is also noteworthy that the presence of **3mU**_−1_ in the "pATSer-GAAA-tetra-loop" substrate rescued cleavage at +1 using the U_248_ RPR variant. Together these data do not support *cis* WC/WC pairing between U_−1_ and A_248(wt)_ in wild type *Eco* RPR. In this context we emphasize that substituting A_248(wt)_ with U influenced the structure of the RPR, in particular in the P18 region, which has a role in connecting the S- and the C-domains. The P18 loop interacts with P8 and disruption of this interaction affects cleavage efficiency of both pre-tRNAs and model hairpin loop substrates^47–51^. Hence, this structural change in the RPR might therefore have an impact on the catalytic performance of the U_248_ variant, both with respect to site selection and rate of cleavage; however, again this would be substrate dependent. This would be in keeping with a perturbed coupling (i.e. induced fit, see e.g. Ref.^15^) between a productive TSL-TBS interaction and events at the cleavage.

Furthermore, in *E. coli* as well as in other bacteria a U is the most frequently (≈60%) occurring nucleobase at −1 in pre-tRNA 5ʹ leaders^24,25,27,28^. This also applies to the archaea *Pyrococcus furiosus* (65% U_−1_), which as *E. coli* possess a type A RPR and an A at the corresponding position to A_248_^9,12,22^ (Fig. 1B). As discussed above, there is limited support for *cis* WC/WC pairing between U_−1_ and A_248(wt)_ in wild type *Eco* RPR. High GC-content bacteria such as *Mycobacterium tuberculosis* (and other mycobacteria; see Fig. 1B) and *Neisseria meningitides* carry type A RPRs with A_248(wt)_ (*E. coli* numbering). In these bacteria, C at −1 is the most frequently occurring nucleobase, while U_−1_ is present in ≈13% and ≈32% of the pre-tRNAs, respectively^27,28^. This argues against formation of *cis* WC/WC pairing between N_−1_ and A_248(wt)_ for the majority of pre-tRNAs in these bacteria.

In conclusion for the majority of pre-tRNAs (and model substrates), A_248_ does not interact with N_−1_ via *cis* WC/WC pairing. However, given that RNase P processes other RNA transcripts, including mRNAs^2^, we cannot completely exclude the possibility that A_248(wt)_ is engaged in *cis* WC/WC pairing with these substrates. In this context we also have to consider that our experiments were performed without the C5 protein and hence the presence of C5 might have an impact given that C5 interact with residues upstream of N_−1_ (see above^41,42^). We propose that the structural architecture of the "active site" is flexible and varies dependent on the identity of the nucleobases at and near the cleavage site and their potential to interact with chemical groups in the RPR. This flexibility is also predicted to depend on the interaction between the pre-tRNA TSL-region and its binding site (TBS) in the RPR S-domain (see above) as well as the RCCA-RPR interaction^15,24,25,30,37,44,46^.

### Structural architecture and Me(II)-binding near the cleavage site

RNase P mediated cleavage depends on Me(II)-ions, which are involved in activating the water molecule that acts as the nucleophile, substrate interaction and folding of the RPR^43,52^. On the basis of correctness and rate of cleavage available data suggest that Mg^2+^ is the preferred ion. Perreault and Altman^53,54^ suggested that binding of Mg^2+^ at the junction between the single stranded 5ʹ leader and the amino acid acceptor stem involves the two 2ʹ hydroxyls at positions −1 and −2 forming a productive complex that acts as the true RNase P substrate, see also^25,38,39,46,55,56^. In RNA the structural topology of Me(II)-binding sites affects both binding affinity and positioning of the Me(II)-ion. This is evident from lead(II)-induced cleavage studies of yeast tRNA^Phe^ and *Eco* RPR^15,57–59^. For model substrates, introduction of U_+1_ (or C_+1_) in pATSerUG (or pATSerCG) affects lead(II)-induced cleavage at the cleavage site such that the frequency of cleavage 5ʹ of N_+1_ increases more than when a purine is present at +1^60^. Similarly, substituting the 2ʹOH at -2, −1 and "C_+74_" in a model hairpin loop model substrate influences Mg^2+^-induced cleavage between −3 and −2^53^. In keeping with this, substituting the N_−1_ 2ʹOH with 2ʹNH_2_ in pATSerUG prevent Pb^2+^-induced cleavage between residue −1 and +1 (not shown). Also, the presence of a 2ʹNH_2_ at N_−1_ in pATSerUG (and pATSerCG) result in a shift of cleavage from −1 to +1 with increasing pH^38–40,44^; this report. The pKa for 2ʹNH_2_ is 6.0–6.2 (determined by NMR-spectroscopy using a dinucleotide)^61,62^. Therefore the 2ʹNH_2_ at −1 in pATSer**am**UG is most likely protonated at lower pH. As a consequence, this results in a positive charge at the +1 cleavage site, which interferes with cleavage at +1, causing the cleavage to shift to −1^38,39^. With increasing pH, the 2ʹNH_3_^+^ becomes deprotonated, resulting in cleavage at +1. The pH dependent shift of cleavage from −1 to +1 (i.e., de-protonation of the 2ʹNH_3_^+^ at −1) is also dependent on the structure of the N_+1_/N_+72_ base pair^40^; this report. The data presented here using the 2ʹNH_2_ substituted substrates suggest that the identity of residue 248 in the RPR also influences the pH dependent shift from −1 to +1, in particular with respect to U_248_. However, we also observed a shift in the pH dependence for G_248_ when the structure of the N_+1_/N_+72_ base pair was altered. Given that A_248(wt)_ is in close proximity to the cleavage site^18^ these data are consistent with a model where changes of the structural architecture at and near the cleavage site in the RPR-substrate complex (see above) affect the charge distribution. As a consequence, this influences the positioning of the Mg^2+^ that activates the water that acts as the nucleophile resulting in a shift of the phosphorus to be attacked^31,43^; for an alternative rational see^25^.

### Proposed function of the well-conserved residue A_248(wt)_ in wild type RPR and base stacking to prevent unspecific hydrolysis

**Figure 9:**
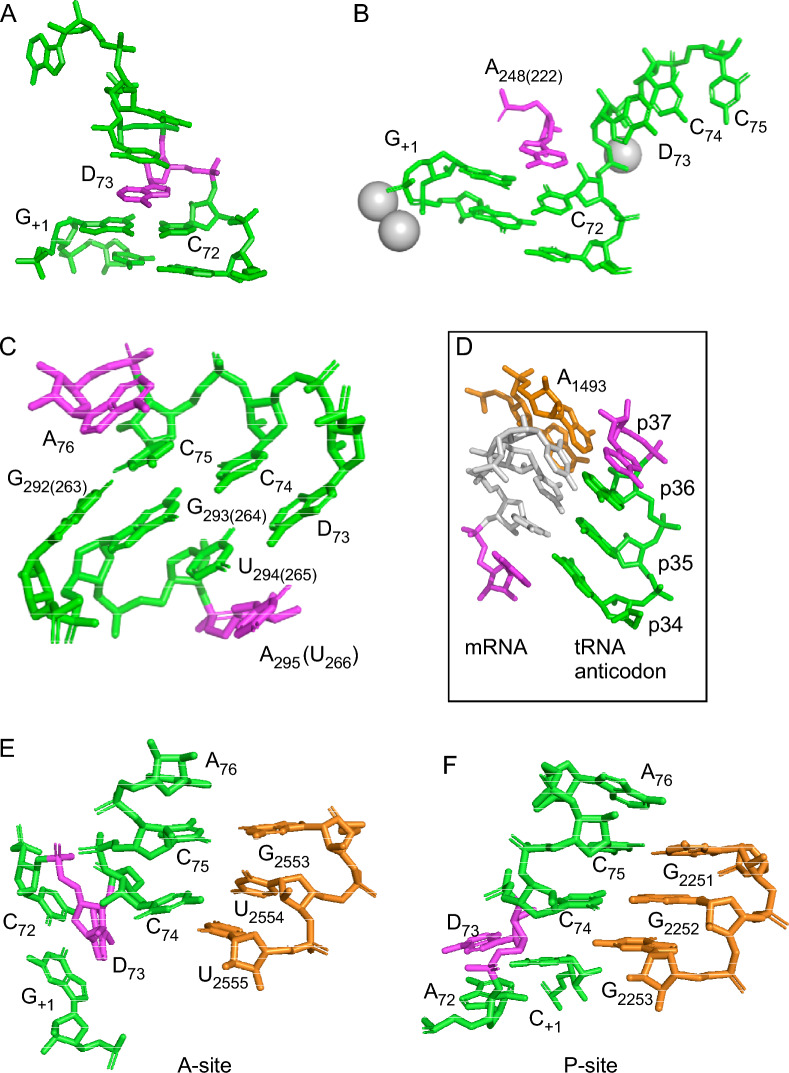
Illustration of base stacking. (**A**) Stacking of the discriminator base, D_+73_ (in magneta), on the G_+1_/C_+72_ base pair in the crystal structure of tRNA^Phe^ (PDB code 1EVV)^64^. (**B**) Stacking of residue A_248_ (in magenta and *E. coli* numbering, Fig. 1) on the tRNA^Phe^ G_+1_/C_+72_ base pair (in green) in the crystal structure of the RNase P-tRNA^Phe^ complex (PDB code 3Q1R)^18^. Grey spheres represent Me(II)-ions. (**C**) Stacking and the RCCA-RPR interaction (in green) in the crystal structure of the RNase P-tRNA^Phe^ complex (PDB code 3Q1R)^18^. Stacking residues in magenta. D_73_ corresponds to the discriminator base at position +73 in tRNA^94^ while the RPR numbering refers to *E. coli* numbering (Fig. 1). Note that A_295_ in *E. coli* corresponds to U_266_ in *T. maritima* RPR^18^. Stacking residues, the tRNA 3ʹ terminal A_76_ and the RPR residue, are marked in magenta. (**D**) Codon-anticodon interaction in the ribosomal A-site where residues in magenta stack as shown in the figure. p34–p37 correspond to positions in the tRNA anticodon loop. Gray residues represent the codon and residues marked in orange residues correspond to A1492 and A1493 in 16S rRNA (PDB code 2J02)^80^. (**E**,**F**) Stacking interactions in the ribosomal peptidyl transfer center, panel E (A-site) and panel F (P-site) as indicated. Orange residues correspond to rRNA residues interacting with the tRNA, green residues refer to tRNA and the tRNA discriminator base is highlighted in magenta (PDB code 5IBB)^79^. The images were created using PyMOL (Schrödinger, LLC).

In the RNase P tRNA crystal structure, which represents the post-cleavage stage, A_248(wt)_ stacks on top of the tRNA G_+1_/C_+72_ base pair and presents the Hoogsteen surface facing the G_+1_ and the tRNA 5ʹ end (Fig. 9A)^17^; see also Refs^20,63^. The importance of the A_248(wt)_ Hoogsteen surface for substrate interaction has been implicated on the basis of nucleotide analogue-modification interferences studies^32^. However, we provided data suggesting that the Hoogsteen surface of A_248(wt)_ is not engaged in pairing with N_−1_, at least not in the case of pMini3bp substrates^31^. This raises the question about the role and function of A_248(wt)_. The structure of yeast tRNA^Phe^ reveals that the discriminator base at position +73 stacks on top of the G_+1_/C_+72_ pair (Fig. 9B)^64^. As such, the discriminator base acts as a hydrophobic cap that restricts access of bulk H_2_O to the terminal base pair^65,66^. Binding of pre-tRNA to the RPR results in formation of the RCCA-RNase P RNA interaction where the discriminator base pairs with residue U_294_^18,27,34^. In the RNase P-tRNA complex A_248(wt)_ stacks on the G_+1_/C_+72_ base pair by occupying the position that the discriminator base has in free tRNA (Fig. 9A,B). This contributes to anchor the substrate to the RPR^18,20,63^. In addition, we propose that the A_248(wt)_ stacking on G_+1_/C_+72_ prevents water from accessing the hydrophobic amino acid acceptor stem and potential unspecific hydrolysis of the tRNA after cleavage. We foresee that this also occurs prior to cleavage of the pre-tRNA and the recent cryoEM structures of *Eco* RNase P in complex with pre-tRNA support that this is indeed the case^63^. In this context the stacking free energy for A would be more favorable, followed by G, C and U^67^. Moreover, considering the activation energy (E_a_), our findings indicated that the trend is A_248(wt)_ < G_248_ < C_248_ < U_248_ with A_248(wt)_ having the lowest activation energy barrier (Table 4). These data provide reasons to why A_248(wt)_ in bacterial RPR is well conserved.

We also note that the E_a_ value for cleavage of pATSerUG with A_248(wt)_ was determined to be 12 kJ/mole (Table 4), which is two- to three-fold lower than for cleavage of pre-tRNA^Tyr^Su3, both with and without the RNase P protein C5^68^. This difference could depend on substrate and/or reaction conditions. In pre-tRNA^Tyr^Su3 both the discriminator base (A_+73_) and the first 3ʹ C (C_+74_) pair with U_−1_ and G_−2_ in the 5ʹ leader, respectively, rendering A_+73_ and C_+74_ less accessible for interacting with RPR, i.e. formation of the "RCCA-RPR interaction" (see above^13^), compared to pATSerUG (Fig. 3). Also, here the experiments were performed at high Mg^2+^ and at a lower pH than in our previous study^68^, which are also factors to consider.

To conclude, in addition to its contribution to anchor the substrate^18,20,63^ we suggest that the function of A_248(wt)_ is to replace the tRNA discriminator base and prevent access of water that would lead to unspecific hydrolysis/cleavage of the pre-tRNA in the RNase P-substrate complex. *Saccharomyces cerevisiae* RPR lacks an A at the position corresponding to *Eco* RPR A_248(wt)_. Interestingly, in the cryo-EM structure of *S. cerevisiae* RNase P in complex with pre-tRNA the 5ʹ leader residues A_−1_ and A_−2_ stack on top of the tRNA G_+1_/C_+72_ pair forming a hydrophobic cap^19^. According to our proposal this would also prevent unspecific hydrolysis/cleavage of the pre-tRNA. Given that POP5 amino acid residues are also positioned close to the G_+1_/C_+72_ pair these might also contribute to prevent access of H_2_O and unspecific hydrolysis/cleavage (see also below).

### Prevention of unspecific hydrolysis in PRORP

Like RNase P, proteinaceous PRORPs cleave the 5ʹ leader of pre-tRNAs and recent data show that the N_−1_ identity also influences cleavage by PRORPs both with respect to cleavage site recognition and rate of cleavage^69–71^. The crystal structures of PRORP1 and PRORP2 are available^72,73^; for a cryo structure see^74^, whereas the structure of PRORP in complex with its pre-tRNA substrate is not. Structural and mechanistic studies suggest that D474 and D475 coordinate Me(II) in the PRORP1 active site. Given the similarities between RNA and protein-based RNase P activities, i.e., the need to cleave pre-tRNAs correctly and prevent unspecific hydrolysis, it is likely that stacking on top of the N_+1_/N_+72_ base pair is also present in the PRORP-pre-tRNA complex. Candidates to act as a hydrophobic cap during the PRORP catalyzed reaction might be aromatic amino acids such as W478 and F500, which both are positioned close to the Me(II)-ion in the active site. Another possibility is that the pre-tRNA discriminator base keeps its position and stacks on top of the N_+1_/N_+72_ pair (and/or residues in the pre-tRNA 5ʹ leader, see above) in the PRORP-substrate complex as observed in other protein-tRNA complexes (see below). It will be interesting to determine whether this is the case and, if so, how access of water to the "inside" of the hydrophobic amino acid acceptor stem is prevented in the PRORP-substrate complex.

### Base stacking and prevention of unspecific hydrolysis of RNA

Crystal structures of amino-acyl-tRNA synthetase-tRNA complexes (such as ArgRS-tRNA_Arg_ and MetRS-tRNA_Met_), EF-Tu-tRNA^Phe^, the CCA adding enzyme in complex with a tRNA mimic and tRNA bound to the ribosome show that the discriminator base at +73 stacks on the G_+1_/C_+72_ pair in a similar way as shown in Fig. 9A (see also E,F)^75–79^. In all these examples the discriminator is a purine. Moreover, inspection of the RCCA-RNase P RNA interaction in the RNase P-tRNA crystal structure reveals that U_266_ stacks on the A_+73_/U_265_ base pair, while the 3ʹ terminal A_+76_ stacks on the C_+75_/G_263_ base pair (Fig. 9C; note that the *T. maritima* residues G_264_, U_265_ and U_266_ correspond to G_292_, U_294_ and A_295_ in wild type *Eco* RPR, see Fig. 1)^18^. A similar type of stacking can also be observed in the ribosomal A- and P-sites both in the case of tRNA and mRNA interaction as well as with respect to the pairing between C_74_ and C_75_ and rRNA (Fig. 9D–F)^79–81^. Together this further emphasizes the importance of stacking. It is conceivable that a function of this "type" of base stacking is to prevent the access of water to functionally important base pairing interactions, and thereby ensuring high fidelity during RNA processing and decoding of mRNA.

## Materials and methods

### Preparation of substrates and RPR

The tRNA^Ser^Su1 precursor (pSu1) N_−1_ variants were generated as run-off transcripts using T7 DNA-dependent RNA polymerase and PCR-amplified templates as described elsewhere^33,82^. The model hairpin loop substrate N_−1_ series (pATSer, pATSer-GAAA-tetra loop and pMini3bp) were purchased from Thermo Scientific Dharmacon, USA. The substrates were [γ-^32^P]-ATP 5ʹ end-labeled and gel-purified followed by overnight Bio-Trap extraction (Schleicher and Schuell, GmbH, Germany; Elutrap in USA and Canada) and phenol–chloroform extraction as described elsewhere^15,31^.

The construction of the gene encoding *Eco* RPR_G248_ was recently reported^31^, while the C_248_ and U_248_ variants behind the T7 promoter were generated following the same procedure as outlined elsewhere using the wild type *Eco* RPR_A248(wt)_ gene as template and appropriate oligonucleotides^12,31,83,84^. The RPRs were generated as run-off transcripts using T7 DNA-dependent RNA polymerase and PCR-amplified templates^31,82^.

### Structural probing of the Eco RPR variants

The *Eco* RPR variants were 3ʹ-end labeled with [^32^P]pCp and structurally probed using Pb^2+^ and RNase T1 under native conditions as described elsewhere^31,34,35,45,85^. Briefly, approximately 2 pmols of labeled RPR in 10 µl was pre-incubated for 10 min at 37 °C in 50 mM Tris–HCl (pH 7.5), 100 mM NH_4_Cl and 10 mM MgCl_2_ together with 4 µM of the unlabeled corresponding RPR. Cleavage was initiated by adding freshly prepared Pb(OAc)_2_ to a final concentration of 0.5 mM and the reaction was stopped after 10 min. In the digestion with RNase T1, the RPR was pre-incubated as described above. One unit of RNase T1 was added followed by incubation on ice for 10 min. The reactions were stopped by adding two volumes of stop solution (10 M urea, 100 mM EDTA). The products were analyzed on 8% (w/v) denaturing polyacrylamide/7 M urea gels.

### Cleavage assays and determination of k_app_

The cleavage reactions were conducted in buffer C [50 mM 4-morpholineethanesulfonic acid (MES) and 0.8 M NH_4_Cl (pH 6.1)] at 37 °C and 800 mM Mg(OAc)_2_. The RPRs were pre-incubated at 37 °C in buffer C and 800 mM Mg(OAc)_2_ for at least 10 min to allow proper folding before mixing with pre-heated (37 °C) substrate. In all the experiments the concentrations of substrates were ≤ 0.02 µM, while the concentrations of the RPR variants were as indicated in Table and Figure legends. The reactions were terminated by adding two volumes of stop solution (see above). The products were separated on 25% (w/v) polyacrylamide/7 M urea gels.

Cleavage of pATSerU_am_G derivatives at 37 °C was performed in buffer C and 800 mM Mg(OAc)_2_ at pH 5.2, pH 6.1 and pH 7.2^39,40^.

The rate constant k_app_ was determined under single-turnover condition at 800 mM Mg^2+^ in buffer C. The concentrations of *Eco* RPR variants used to generate the data are specified in the respective Table legends. The concentrations of pSu1 (precursor-tRNA^Ser^Su1^34^) and model substrates^15,31,34^ were ≤ 0.02 μM (see also the main text). For rate calculations, we used the 5ʹ cleavage fragment as a measure of product formed. In each assay, the time of incubation was adjusted to ensure that the velocity measurements were in the linear range (typically ≤ 10%, but never exceeding that 40% of the substrate had been consumed). Each k_app_ value is reported as a mean ± deviation of this value, which was calculated using data (six time points) from at least three independent experiments.

### Determination of the kinetic constants k_obs_, k_obs_/K^sto^ and K^sto^

The rate constants k_obs_ and k_obs_/K^sto^ were determined under saturating single-turnover conditions at pH 6.1 (where cleavage is suggested to be rate limiting) and 800 mM Mg^2+^ using pATSerUG, as described elsewhere, e.g.^37^. Under these conditions we have argued elsewhere that K^sto^ ≈ K_d_ in the *Eco* RPR-alone reaction^12,30,31,86,87^. The final concentrations of the different RPR variants were between 0.8 and 6.4 µM; the concentration of the pATSerUG substrate was ≤ 0.02 μM. To ensure that the experiments were done under single-turnover conditions, the lowest concentration of RPR was > 10 times higher than the concentration of the substrate. For the calculations we used the 5ʹ cleavage fragment, and the time of cleavage was adjusted to ensure that the velocity measurements were in the linear range (see above). To be able to compare with our previously published data, k_obs_ and k_obs_/K^sto^ were obtained by linear regression from Eadie-Hofstee plots as described elsewhere^12,30,31,37,88,89^. Each value is an average of at least three independent experiments and is given as a mean ± the deviation of this value.

## Data Availability

The datasets used are available from the corresponding author.
